# Linkage Mapping and Comparative Transcriptome Analysis of Firmness in Watermelon (*Citrullus lanatus*)

**DOI:** 10.3389/fpls.2020.00831

**Published:** 2020-06-16

**Authors:** Lei Sun, Yushu Zhang, Haonan Cui, Lupeng Zhang, Tongyun Sha, Chaonan Wang, Chao Fan, Feishi Luan, Xuezheng Wang

**Affiliations:** ^1^College of Horticulture and Landscape Architecture, Northeast Agricultural University, Harbin, China; ^2^Key Laboratory of Biology and Genetic Improvement of Horticulture Crops (Northeast Region), Ministry of Agriculture, Harbin, China

**Keywords:** firmness, watermelon, mapping, QTL, transcriptome

## Abstract

Watermelon fruit texture and quality are determined by flesh firmness. As a quality trait, flesh firmness is controlled by multigenes. Defining the key regulatory factors of watermelon flesh firmness is of great significance for watermelon genetic breeding. In this study, the hard-flesh egusi seed watermelon PI186490 was used as the male parent, the soft-flesh cultivated watermelon W1-1 was used as the female parent, and 175 F_2_ generations were obtained from selfing F_1_. Primary mapping of the major genes controlling center flesh firmness was achieved by bulked-segregant analysis (BSA)-Seq analysis and molecular marker technology. Finally, major genes were delimited in the physical interval between 6,210,787 and 7,742,559 bp on chromosome 2 and between 207,553 and 403,137 bp on chromosome 8. The content of each cell wall component and hormone was measured, and comparative transcriptome analysis was performed during fruit development in watermelon. The protopectin, cellulose, hemicellulose, indole-3-acetic acid (IAA) and abscisic acid (ABA) contents were measured, and paraffin sections were made during the three fruit developmental stages. The results revealed that protopectin, celluloses, and hemicelluloses exhibited similar trends for flesh firmness, while the IAA and ABA concentrations continued to decrease with fruit ripening. Paraffin sections showed that PI186490 cells were more numerous, were more tightly packed, had clearer cell wall edges and had thicker cell walls than W1-1 cells at every developmental stage. Comparative transcriptome analysis was conducted on RNA samples of flesh during fruit development and ripening in W1-1 and PI186490. The results from the localization interval transcriptome analysis showed that Cla016033 (DUF579 family member), which may influence the cell wall component contents to adjust the flesh firmness in watermelon fruit, was different in W1-1 and PI186490 and that Cla012507 (MADS-box transcription factor) may be involved in the regulation of fruit ripening and affect the hardness of watermelon fruit.

## Introduction

Watermelon (*Citrullus lanatus* (Thunb.) Matsum & Nakai) is an annual herb that belongs to the gourd family and is an important horticultural crop worldwide. Watermelon fruit quality mainly includes the commodity quality, sensory quality, and nutritional quality. Flesh firmness, as an important attribute of the watermelon sensory quality, is an important index for measuring fruit commerciality. Flesh firmness affects taste, as increasingly hard flesh results in reduced juice and poor flavor (Harker et al., 2003), while flesh that is too soft has no resistance to storage and a short shelf life (Risse et al., 1990).

A change in fruit hardness is a common physiological phenomenon in nature and is mainly caused by changes in flesh cell structure and material, the degradation of cell wall material in the flesh and the degradation of pectin, cellulose, and hemicellulose structures, which initiates fruit softening. There is a series of complex and comprehensive changes in the structure of cellulose and pectin components and in the content of substances in cells, as protopectin decomposes to form soluble pectin and pectinic acid, which results in decreased firmness (Chen and Zhang, 1998). The degradation of cell walls causes fruit to soften (Basanta et al., 2013; Brahem et al., 2017), and cellulose degradation leads to cell wall disintegration and fruit softening (Brummell et al., 2004). In the study of peach fruit hardness, the hardness of peach fruit was positively correlated with the contents of propectin and cellulose and negatively correlated with the content of soluble pectin (Hu et al., 2007). Liu et al. (2013) suggested that the pectin and crude fiber contents in fruits were the main reason for the difference in flesh firmness between wild watermelon and cultivated watermelon and speculated that the difference in the synthesis and metabolic pathway of pectin and crude fiber in the evolution of watermelon might be the fundamental reason for differences in flesh firmness. The contents of propectin, cellulose, hemicellulose, and covalent binding pectin increase with increasing watermelon flesh hardness, while the content of water-soluble pectin and ionic pectin increase with decreasing watermelon flesh hardness (Gao, 2018). In terms of hormones, abscisic acid (ABA) is also involved in the formation of fruit texture, and the fruit cell wall is usually embedded with an interwoven network of glycoproteins, cytoplasmic membrane microfibers, and xyloglucan. Studies have shown that ABA plays a role in fruit softening The fruit pectin content was significantly higher, shelf life was prolonged, and flesh was harder and more pliable in SLNCED1-RNAi transgenic fruits than in wild-type fruits (Sun et al., 2012b). Indole-3-acetic acid (IAA) plays an important role in fruit setting and early development, while it plays the opposite role in fruit ripening (Sundberg and Stergaard, 2009). Many genes are positively regulated during fruit ripening but suppressed by IAA, including SHATTERPROOF, a gene in strawberry that encodes an MADS-box transcription factor that regulates maturation (Daminato et al., 2013). In addition, the degradation of the cell wall-plasma membrane leads to the softening of fruit, which is related to a significant decrease in the glycoprotein and pectin contents and the remodeling of their permutations (Agata et al., 2018).

As with most fruit quality traits, the watermelon flesh hardness trait is a typical quantitative trait that is inherited, and the change in flesh hardness is the result of genotype and environment, which may be jointly regulated by numerous genes and metabolic networks (Brummell and Harpster, 2001). Juarez et al. (2013) used single nucleotide polymorphism (SNP) markers to construct a genetic linkage map of an F_2_ isolated population, including 19 linkage groups, and located a major quantitative trait locus (QTL) related to watermelon flesh firmness on the 9th linkage group. Liu et al. (2014) located genes of edge flesh hardness and center flesh hardness on the 9th linkage group of watermelon in an F_2_ isolated population by resequencing two parents. Lu et al. (2016) located genes of edge flesh hardness on the 4th, 6th, and 8th linkage groups. The simple sequence repeat (SSR) marker BVWS00954 (a primer on chromosome 6) was identified to be closely linked to the gene controlling the firmness of watermelon flesh (Gao et al., 2016). Gao (2018) located the gene controlling watermelon flesh firmness in a 4.7 Mb physical interval of chromosome 6.

Watermelon transcriptome research started relatively late; Wechter et al. (2008) used 832 expressed sequence tags (ESTs) in a cDNA library to study gene expression in the process of watermelon fruit development, of which 211 ESTs were differentially expressed and were mainly involved in ethylene biosynthesis, transcriptional regulation, pathogen and rib forced response and carotenoid biosynthesis. A total of 3023 differentially expressed genes (DEGs) were identified during the development and ripening of watermelon fruits, mainly encoding genes related to pigment and sweetness metabolites (Guo et al., 2011). Guo et al. (2015) identified key genes in glucose metabolism and accumulation, flesh carotene biosynthesis and metabolism, flesh texture, and the ethylene biosynthesis and signal transduction pathway by comparing the transcriptome of wild watermelon and cultivated watermelon fruits at different development stages. Zhu et al. (2017) conducted comparative transcriptional analysis of two watermelon fruit samples at different development stages and identified DEGs that were mainly involved in biological processes related to sugar and cell wall metabolism, carotenoid biosynthesis, and plant hormone metabolism pathways. Gao (2018) conducted transcriptional analysis on the watermelon flesh hardness of near-isogenic “HWF” at development stages and found that there were many DEGs related to fruit texture, including pectinesterase, glycosyl transferase, cellulose synthesis, polygalacturonase, and xyloglucan endotransglycosylase.

Flesh firmness is an important factor in determining the flesh quality of watermelon, which is a complex quantitative trait controlled by multiple genes. The traditional physiological and genetic research methods have limitations in revealing the genetic mechanism and the molecular regulatory mechanism of complex traits. Further identification of the key regulatory factors of watermelon flesh hardness can provide theoretical and technical support for watermelon quality breeding. In this study, We investigated the inheritance of the watermelon center flesh hardness gene in the F_2_ population of “W1-1” (soft-flesh fruit) × “PI186490” (hard-flesh fruit). We identified candidate genes on chromosome 2 and chromosome 8 associated with watermelon center flesh hardness through BSA and transcriptome analysis. We also identified the changes in the IAA and ABA contents and in the cell wall materials during watermelon development and completed cell observation by paraffin sectioning.

## Materials and Methods

### Plant Materials

**FIGURE 1:**
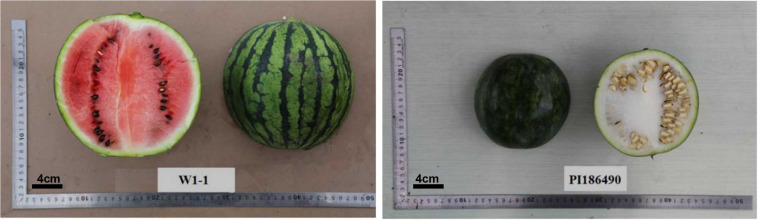
Soft-flesh cultivated watermelon W1-1 was used as the female parent and hard-flesh egusi seed watermelon PI186490 was used as the male parent.

The female material W1-1 is a homozygous cultivated watermelon strain from the Laboratory of Melon and Watermelon, Northeast Agricultural University. The firmness measured by the GY1 hardness tester was 2 × 10^5^ kg/cm^2^. The male material PI186490 is the homozygous egusi seed watermelon strain provided by Angela R. Davis who works at the U.S. Department of Agriculture. The firmness measured by the GY1 hardness tester was 15 × 10^5^kg/cm^2^ (Figure 1).

For the genetic analysis study, phenotypic data of center flesh hardness were collected in three controlled-environment at Xiangyang farm in Harbin, China in 2017, 175 F_2_ individual were grown in April 25, 2017 (G1), 734 F_2_ individual were grown in May 25, 2017 (G2), and 388 F_2_ individual were grown in June 25, 2017 (G3), respectively, and each test planted parents lines and F_1_. Flowers were hand-pollinated and tagged to record the number of days after pollination (DAP). For parental materials, at each of three stages (pollinated 7, 21, and 35 days), 10 fruits were selected for uniformity of size and were randomly divided into two groups. In one group, the fruit was evenly divided into two halves in the longitudinal direction, and a sample size of 2 cm × 2 cm × 2 cm was collected from the center of the half of the fruit for the texture profile analysis (TPA) test. The center of the other half of the fruit was collected and immediately frozen in liquid nitrogen, delivered rapidly to the laboratory and stored at −80°C. In the other group, the center of the fruit was collected in FAA solution. For F_2_ individual plants at the mature stage, fruits were cut in the longitudinal direction, and the center fruit hardness was measured by a GY1 fruit hardness tester. Three points were taken from each side, a total of six experimental data points were collected, and the data with large differences were removed.

### BSA and Mapping Strategy

Leaves were selected from each plant, and DNA was extracted by the CTAB method with slight modifications. Based on the fruit firmness of watermelon, 20 individual lines with very hard fruit and 20 individual lines with very soft fruit from the F_2_ population were selected and grouped as two bulks for the BSA. The bulks were sequenced on an Illumina HiSeq^TM^ 2500 platform (over 10-fold genome coverage) at BGI (Shenzhen, China). After the sequencing results were obtained, we used an in-house bioinformatics platform for analysis of the BSA data. After the reads were checked for quality, the clean short reads were aligned to the “97103” reference genome (Guo et al., 2013) with the Burrows–Wheeler Aligner (BWA) software, version 0.6.2 with the default configurations (Li and Durbin, 2009). The reads from two bulks were then aligned, and variants were called for both the bulks against the developed assembly (Koboldt et al., 2013). Single nucleotide polymorphism-index was calculated for all the SNP positions. To determine the candidate regions for watermelon flesh hardness QTL, two parameters, SNP-index and Δ (SNP-index) (Abe et al., 2012) were calculated.

Cleaved amplified polymorphism sequence (CAPS) markers were developed based on previously acquired high-throughput sequencing data of the two parents (W1-1 and PI186490) on the BGI platform. A total of 10 G data was obtained from parents based on 10-fold coverage of the watermelon genome. The reads were filtered to remove all low quality regions by an in-house Perl program and mapped to watermelon reference genome assembly (v1) (Guo et al., 2013) using the BWA software (Li and Durbin, 2009). SAM tools software was used to sort and index map reads with mapping scores of 20 (Li and Durbin, 2009), and on both sides of each candidate SNP locus in W1-1 and PI186490, 500 bp flanking sequences were obtained by SAMtools (*Q* = 20). The candidate SNP loci were transformed into CAPS markers using SNP2CAPS (Thiel et al., 2004). PCR primers in the target chromosomes were designed by BSA sequencing analysis. Primers were designed with Primer Premier 6.0^1^ and synthesized by Sangon Biotech. The primers were named according to the formula: H + number of chromosomes + number of primers. PCR amplification and enzyme digestion were performed as described by Liu et al. (2019).

QTL IciMapping (Version 4.0) software (Meng et al., 2015) was used to construct a linkage map, with a total of 90 individuals and 24 polymorphic CAPS markers. The F_2_ individuals that exhibited the band pattern of the female and male parents were recorded as A and B, respectively, and the patterns of the F_1_ types were marked as H. The results were imported into QTL IciMapping for analysis with a filtering threshold of a limit of detection (LOD) > 2.5 to check the gene locus.

### RNA-Seq Library Preparation and Sequencing

Total RNA from frozen watermelon fruit flesh from every fruit stage was isolated using the RNA plant Plus Reagent Kit (TIANGEN, Beijing, China) according to the instructions (three biological replicates per sample). Purity (OD260/280), concentration and nucleic acid absorption peak of the RNA samples extracted were detected with a Nanodrop Nano Photometer (Implen GmbH, Munich, Germany); RNA integrity was accurately detected using an Agilent 2100 Bioanalyzer (Agilent Technologies, CA, United States). Qualified RNA was used to obtain the library. After the construction of the library, a Qubit2.0 fluorometer was used for preliminary quantification, and the library was diluted to 1.5 ng/μl. Then, the insert size of the library was detected by an Agilent 2100 Bioanalyzer. Once the insert size was in agreement with expectations, qRT-PCR was used for accurate quantification of the effective concentration of the library (the effective concentration of the library was higher than 2 nM) to ensure the quality of the library. Once the library quality was confirmed, Illumina sequencing was performed for different libraries.

### Sequencing Quality Control, Comparison and Data Analysis

After sequencing, the data were filtered, mainly to remove reads with adapters, reads containing N (N means indeterminate base information), and reads of low quality (reads with Qphred ≤ 20 bases accounting for more than 50% of the read length). All of the clean reads were deposited in the NCBI Short Read Archive (SRA) database under the accession number PRJNA477364. TopHat2 (Kim et al., 2015) was used to align the sequences of the clean reads with the watermelon reference genome assembly (v1) (Guo et al., 2013) to obtain the location information in the reference genome or gene and the sequence characteristic information unique to the sequencing samples.

The core of RNA-Seq is the analysis of significant DEGs using statistical methods to compare the DEGs under two or more conditions. Specific genes associated with the conditions were identified, and the biological significance of specific genes was further analyzed. DESeq (Anders and Huber, 2010) was used to identify DEGs with biological duplicates between sample groups. To categorize the DEGs during fruit ripening, we used a stringent value of a false discovery rate (FDR) ≤ 0.05 and an absolute value of log2 ratio ≥1 as the thresholds for identifying significant differences in gene expression between W1-1 and PI186490.

The Gene Ontology (GO) database is a comprehensive database describing gene functions, which can be divided into the categories molecular function, biological process and cellular component. GO terms with padj < 0.05 were considered significantly enriched, and WEGO (Ye et al., 2006) was used for GO functional classification. The Kyoto Encyclopedia of Genes and Genomes (KEGG) database is a comprehensive database integrating genomic, chemical and system functions, and it is conducive to researchers’ studies of genes and their expression information as an overall network. KEGG pathways with padj < 0.05 were considered significantly enriched. As the main public database of pathways (Kanehisa et al., 2008), KEGG provides information on integrated metabolic pathways, including metabolism of carbohydrates, nucleosides and amino acids, and the biodegradation of organic compounds.

The qRT-PCR was performed as described Gao (2018) (three biological replicates per sample).

### TPA Test Method

The TPA of the watermelon cultivars was performed using a texture analyzer (TA-XT2i, SMS Corporation, United Kingdom). The TPA was performed as described by Pan and Tu (2005), and a P/35 probe was used for this test. Each sample was measured at two points and each stage had three biological replicates. The parameters of the determinations were as follows: force induction range = 250 N, deformation 100 = 75%, test speed = 1.0 mm/s, and input starting force = 0.2 N. Six textural parameters, namely, hardness, chewiness, cohesiveness, springiness, resilience, and adhesiveness, were determined from each curve.

### Cellulose, Protopectin, Hemicellulose, IAA, and ABA Contents in Watermelon Fruit

Cellulose, protopectin, and hemicellulose were extracted as described by Chi (2012), neutral detergent reagent (NDR) was used to remove sugar, starch, protein, pectin, and other substances from the sample. The residue is thought to contain mainly insoluble carbohydrates. Cellulose and hemicellulose have different hydrolysis abilities in a dilute acid solution, and therefore, dilute HCl can be used to separate them. Lignin is difficult to hydrolyze in acid, and 72% H_2_SO_4_ can separate it from other residues. Eventually, we obtained the cellulose and hemicellulose contents using the colorimetric method. IAA and ABA were extracted by Suzhou Comin Biotechnology Co. Ltd (Soochow). Three biological replicates per sample.

### Paraffin Sectioning Method

The paraffin sections were made as described Gao et al. (2013); parents in different stages of fruit development were collected and immediately fixed in FAA (a 1:1:18 mixture (v/v/v) of formalin:acetic acid:50% ethanol). After being dehydrated in an ethanol series, infiltrated with xylene and embedded in paraffin wax by conventional methods, samples were sectioned using a microtome. After sectioning, the paraffin was removed and observed. Three biological replicates per sample.

## Results

### Analysis of Watermelon Flesh Hardness Data of the F_2_ Isolated Population

**FIGURE 2:**
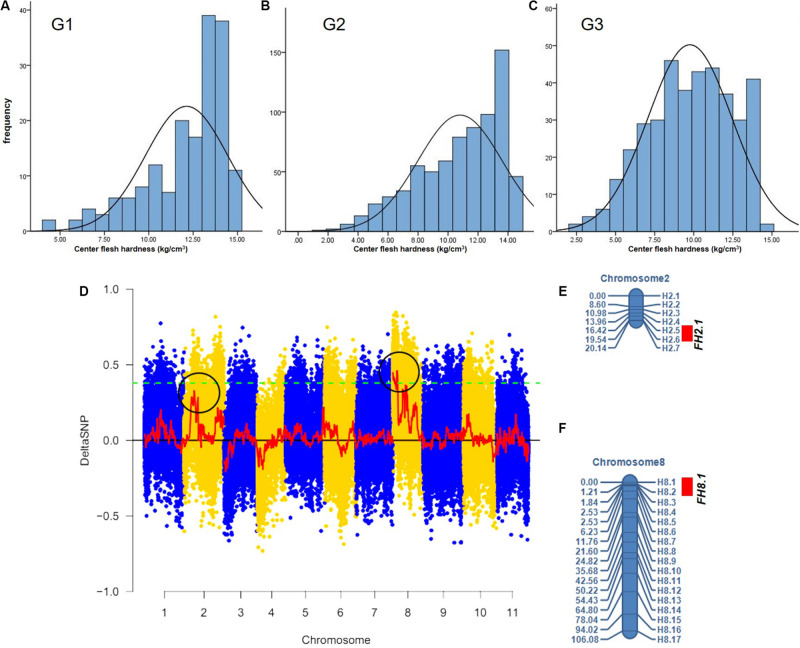
The fruit hardness (FH) genes *FH8.1* and *FH2.1* genes were mapped on chromosome 8 and chromosome 2, respectively. Frequency distribution of fruit hardness in the F_2_ population in different environment **(A–C)**, **(D)** the target genes were mapped on chromosome 8 and chromosome 2 by BSA analysis, **(E)** a Linkage map of chromosome 2 based on 90 F_2_ individuals from G1, and **(F)** a Linkage map of chromosome 8 based on the same 90 F_2_ individuals from G1.

The center fruit hardness of the three F_2_ population was surveyed. SPSS 22.0 software was used to plot the histogram of the frequency distribution, and the results are shown in Figures 2A–C and Table 1. The female parent “W1-1” had a fruit hardness of 2 × 10^5^ kg/cm^2^ at the center of the fruit, and the parent “PI186490” had a fruit hardness of 15 × 10^5^ kg/cm^2^ at the center of the fruit, thus revealing the obvious differences in the center fruit hardness of the two parents. The flesh hardness of each individual in the three F_2_ population was between the tested parents, showing a continuous distribution; the overall trend characterized by obvious partial separation of the distribution variation range was relatively large, indicating that there were major genes controlling the firmness of watermelon center flesh.

**TABLE 1:** Phenotypic means and range of center flesh hardness of W1-1, PI186490, their F_1_ and F_2_ from three experiments (G1, G2, and G3).

**Evn^a^**	**W1-1**	**PI186490**	**F_1_**	**F_2_population**		
	**(kg/cm^2^)**	**(kg/cm^2^)**	**(kg/cm^2^)**	**(kg/cm^2^)**		
	**Mean ± SD^b^**	**Mean ± SD**	**Mean ± SD**	**Mean**	**Range**	**SD**
G1	2 ± 0	15 ± 0	12.175 ± 0.287	12.130	4.4–15.0	2.319
G2	2 ± 0	15 ± 0	13.100 ± 0.849	10.799	2.0–14.8	2.816
G3	2 ± 0	15 ± 0	13.500 ± 0.408	9.750	2.1–14.3	2.696
CE^c^	2 ± 0	15 ± 0	12.675 ± 0.854	10.821	2.0–15.0	2.798

*^*a*^Environment. ^*b*^Standard deviation. ^*c*^Combined environment (average from the three different environments).*

### Localization of Watermelon Center Flesh Firmness Genes

The F_2_ generation of the secondary isolated population was generated by the soft-flesh watermelon W1-1 and hard-flesh watermelon PI186490. Phenotypic identification of flesh hardness was carried out on individual F_2_ generation secondary isolated populations. Twenty individual plants with extreme traits were selected to construct hard-flesh and soft-flesh pools, respectively, the bulks were sequenced on an Illumina HiSeq^TM^ 2500 platform at BGI (Shenzhen, China). A total of 19.32 Gb of raw reads data was generated, and the sequencing depth of each pool was approximately 24×. After the reads were checked for quality, the clean short reads were aligned to the “97103” reference genome (Guo et al., 2013) with the BWA software, version 0.6.2 with the default configurations (Li and Durbin, 2009). Subsequently, the SNP index was calculated from two bulks using QTL-seq pipeline based on the stringent selection criteria, total 586,429 SNPs were identified on all 11 chromosomes. Next, the Δ(SNP-index) was calculated and plotted with the threshold of Δ(SNP-index) value was 0.4 to obtain a SNP differential locus distribution map of watermelon center flesh hardness stripes (Figure 2D). The results showed that there was a genome region on chromosome 8 with DSNP values larger than the threshold values, and the physical distance was 0–4,130,000 bp. Moreover, there was a genomic region on chromosome 2 with DSNP values very close to the threshold value, and the physical distance was 7,047,502–12,282,807 bp, indicating that genes that control watermelon fruit center hardness were present on chromosomes 8 and 2. A total of 24 primers (17 on whole chromosome 8 and 7 in the target region of chromosome 2) were used for the gene localization of 90 F_2_ individuals from G1. A linkage map including these markers was constructed using QTL IciMapping (Version 4.0) software (Figures 2E,F). Finally, a locus (*FH8.1*) closely linked to the watermelon center fruit hardness trait was mapped between the CAPS markers H8.1 and H8.2, from 207,553to 403,137 bp, where the physical distance was approximately 195 kb, the LOD value was 4.6616 and the proportion of variation explained (PVE) value was 24.4416%. A locus (*FH2.1*) closely linked to the watermelon fruit center hardness trait was mapped between the CAPS markers H2.5 and H2.6, from 6,210,787 to 7,742,559 bp, where the physical distance was approximately 1.53 Mb, the LOD value was 2.9028 and the PVE value was 14.0475%.

### Comprehensive Evaluation of RNA-Seq Data

To understand the possible molecular synthesis mechanism for differences in watermelon flesh hardness, 18 cDNA libraries were constructed from three key periods of fruit development in W1-1 and PI186490. The data quality was assessed after RNA sequencing, and the comprehensive evaluation of sequencing and assembly is shown in Table 2.

**TABLE 2:** An overview of the RNA-Seq data.

**Sample**	**Raw reads number**	**Clean reads number**	**Total mapped reads (%)**	**GC content (%)^a^**	**Q30 (%)^b^**
W1_1A_1	30,350,020	29,752,031	96.3	44.35	94.66
W1_1A_2	28,454,167	27,931,032	96.56	44.39	95.06
W1_1A_3	22,197,036	21,689,380	96.22	44.40	95.15
W1_1C_1	23,338,097	22,866,696	94.75	44.86	93.99
W1_1C_2	23,854,148	23,302,178	88.78	44.68	94.20
W1_1C_3	21,642,904	21,278,969	93.88	44.87	94.24
W1_1E_1	22,164,996	21,595,832	86.48	44.22	94.02
W1_1E_2	22,591,215	21,780,885	80.28	44.26	93.92
W1_1E_3	22,075,108	21,410,027	80.06	44.20	94.61
Z186A_1	20,101,086	19,697,436	95.24	44.26	94.22
Z186A_2	20,057,401	19,679,166	95.91	43.41	94.46
Z186A_3	21,880,466	21,055,447	95.76	44.35	94.45
Z186C_1	22,893,014	21,905,232	93.84	44.56	94.36
Z186C_2	24,328,898	23,332,115	86.92	44.08	94.07
Z186C_3	22,196,672	21,555,730	92.36	44.23	94.09
Z186E_1	23,907,754	23,054,258	93.13	44.43	93.87
Z186E_2	20,610,118	19,834,825	87.53	44.49	93.37
Z186E_3	20,783,203	20,071,889	83.00	44.39	93.96

*^*a*^GC content (%) are the percentages of G and C in four bases in clean reads. ^*b*^Q30 (%) are the percentages of reads with Phred qualities scores over than 30.*

Transcriptome sequencing of 18 samples was completed, and clean data was obtained for further analysis, with 5.9–8.93 Gb for each sample. After the removal of low-quality reads, an average of 22,152,642 high-quality clean reads was obtained from each sample, accounting for 97.14% of the 22,803,913 raw reads. Clean reads of each sample were sequentially aligned with the designated reference genome, and the alignment efficiency ranged from 80.06 to 96.56%. The Q30 base percentage was 93.37 or higher, and the GC content in all samples ranged from 43.41 to 45.21%.

### Candidate Genes and Analysis

The watermelon genome database^2^ was analyzed, and 10 candidate genes were included in the physical interval of chromosome 8 207,553–403,137 bp (Cla012498, Cla012499, Cla012500, Cla012501, Cla012502, Cla012503, Cla012504, Cla012505, Cla012506, and Cla012507). According to the annotated information of the genes (shown in Table 3), Cla012498 is a TIR-NBS disease resistance-like protein. Cla012499 and Cla012500 were GDSL esterases/lipases, and GDSL esterases/lipases belong to the α/β hydrolytic enzyme family and participate in fruit cuticle regulation in the tomato. Cla012501 is a protein of unknown function. Cla012502 is a ubiquitin-protein ligase involved in the regulation of plant growth and development. Cla012503 and Cla012504 are unknown proteins. Cla012505 and Cla012506 are U4/U6. U5 tri-snRNP-associated protein, and U4/U6. U5 tri-snRNP represents a substantial part of the spliceosome before activation. Cla012507 is a MADS-box transcription factor that participates in the regulation of fruit development, and MADS-box transcription factors have been suggested to be the main switch to regulate the ripening networks of climacteric fruit and non-climacteric fruit. Therefore, Cla012499, Cla012500, Cla012502, and Cla012507 may be related to the metabolism regulation of watermelon flesh firmness. However, combined with the comparative transcriptome analysis of watermelon fruits at five different developmental stages, it was found that only Cla012500 was expressed at 21 DAP and 35 DAP in W1-1, while no other genes were detected at any time. In the watermelon genome database, the expression of Cla012507 in the flesh of 97103 (soft flesh) was always higher than that of PI296341 (hard flesh). There were 92 candidate genes included in the physical interval of chromosome 2 6,210,787–7,742,559 bp, combined with the comparative transcriptome analysis of watermelon fruits at five different developmental stages. Only one gene (Cla016033) was differentially expressed in five stages of fruit development in W1-1 and PI1896490. Cla016033 belongs to the DUF579 family and is a protein of unknown function, while all members of the DUF579 family characterized so far have been described to affect the integrity of the hemicellulose cell wall component xylan. The expression of Cla016033 was significantly different in W1-1 and PI186490; the expression of Cla016033 decreased in W1-1 fruit as the fruit matured, while in PI186490 fruit, the expression of Cla016033 increased first and then decreased, which was consistent with the change in the trend of flesh hardness. The coding sequences (CDS) of Cla016033 did not vary between W1-1 and PI186490 according to Integrative Genomics Viewer (IGV) results, but there was an 11-base (GGCAATCCAAA) insertion at the promoter region of PI186490. The changes in flesh firmness were influenced by hemicellulose in the cell wall and fruit ripeness, and therefore, Cla012507 and Cla016033 could be used as the main candidate genes for watermelon center flesh firmness in further studies.

**TABLE 3:** Annotated information of the genes in target interval of chromosome 8.

**Gene ID**	**Chr**	**Position**	**Description**
Cla012499	8	363,173–367,092	GDSL esterase/lipase
Cla012500	8	349,653–353,102	GDSL esterase/lipase
Cla012501	8	312,195–313,829	Os06g0524700 protein (Fragment)
Cla012502	8	295,281–295,934	Ubiquitin-protein ligase/zinc ion binding protein
Cla012503	8	289,667–289,921	Unknown Protein
Cla012504	8	270,185–270,424	Unknown Protein
Cla012505	8	264,244–268,109	U4/U6.U5 tri-snRNP-associated protein 1
Cla012506	8	246,689–250,474	U4/U6.U5 tri-snRNP-associated protein 1
Cla012507	8	212,501–215,874	MADS box transcription factor

*Cucurbit Genomics Database (CuGenDB) was used.*

### TPA Analysis of PI186490 and W1-1 Fruit During Ripening

**FIGURE 3:**
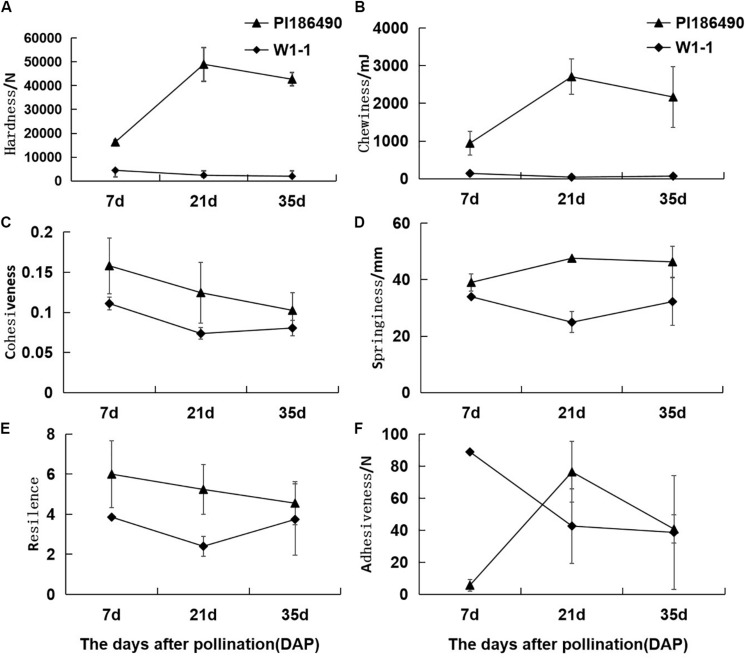
Changes of TPA parameters of PI186490 and W1-1 fruit during ripening. Hardness **(A)**, chewiness **(B)**, cohesiveness **(C)**, springiness **(D)**, resilience **(E)**, and adhesiveness **(F)** were extracted at 7, 21, and 35 DAP. Three individual replicates were used. The bars represent the standard error (SE) (*n* = 3).

During fruit development and ripening, the flesh hardness of PI186490 increased invariably, reached a maximum value at 21 DAP, and decreased in the later period. The flesh hardness of W1-1 was consistently low and declined overall (Figure 3A). Chewiness reflects the energy required to chew the flesh until it was ready to be swallowed. The chewiness and hardness of the flesh of the two watermelon varieties were very closely related, and the chewiness of PI186490 was consistently higher than that of W1-1 and nearly 63 times higher than that of W1-1 at 21 DAP. Fruit with increasingly hard flesh required increasing energy to chew the flesh (Figure 3B). Cohesiveness reflects the size of the binding force between the fruit flesh cells, and resilience reflects the ability of the fruit to quickly recover from deformation after being squeezed. The two watermelon lines showed the same trend of constant decline in cohesiveness and resilience. Moreover, the cohesiveness and resilience of PI186490 were consistently higher than those of W1-1 (Figures 3C,E). Springiness reflects the extent to which a sample recovers after its first compression. The trend of the change in springiness in PI186490 was the same as its firmness: both increased first, then decreased. The springiness and firmness of PI186490 were consistently higher than those of W1-1 (Figure 3D). Adhesiveness reflects the force at which a sample prevents the probe from returning after the sample is first compressed. However, during the test, there were always samples adsorbed by the probe away from the test board, and thus, the measured results were not accurate (Figure 3F).

### Cell Wall Component and Phytohormone Analyses of PI186490 and W1-1 Fruit During Ripening

**FIGURE 4:**
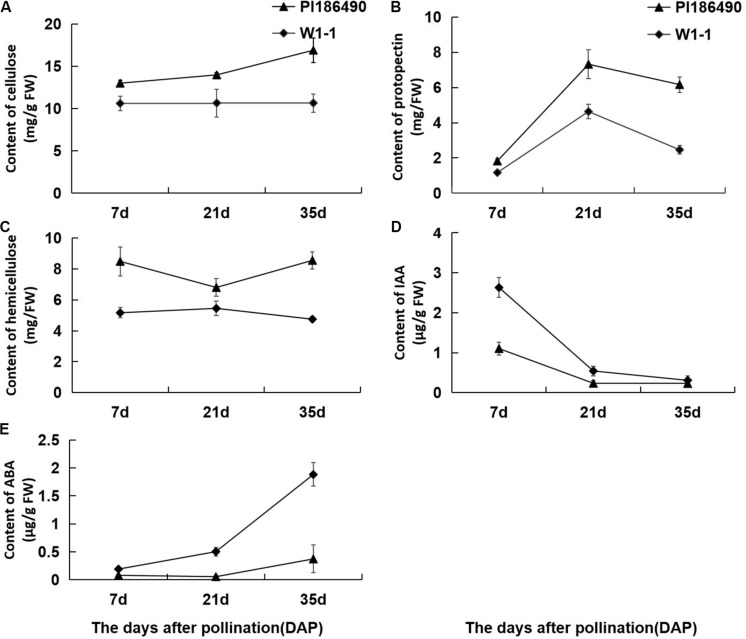
Changes in concentration of cell wall components and phytohormones of PI186490 and W1-1 fruit during ripening. Cellulose **(A)**, protopectin **(B)**, hemicellulose **(C)**, IAA **(D)**, and ABA **(E)** were extracted at 7, 21, and 35DAP. Three individual replicates were used. The bars represent the standard error (SE) (*n* = 3).

Cellulose, pectin and hemicellulose are the main components of cell wall materials, which largely determine the firmness and sensory qualities of watermelon fruit flesh. The dynamic changes in the cell wall components during the development of watermelon fruits are shown in Figures 4A–C: during fruit development and ripening, the concentration of cellulose in PI186490 increased invariably, the trend of W1-1 did not change much, and the cellulose content of PI186490 was nearly 1.6 times higher than that of W1-1 at 35 DAP. Trends in the content of protopectin in PI186490 and W1-1 fruit were similar, with both increasing first before 21 DAP and decreasing at the later period. The changes in the hemicellulose in the two samples were most significant at 35 DAP, indicating a 1.8-fold difference. The content of the cell wall components in PI186490 fruit was higher than that in W1-1 at any evaluated time. These results showed that during the development and maturation of watermelon fruits, the content of cell wall components in PI186490 and W1-1 fruits were significantly different, which played a decisive role in the change in flesh hardness. The content of IAA in W1-1 was significantly higher than that in PI186490 at 7 DAP and it continuously declined until 35 DAP, when the content of IAA was not substantially different between the two samples. These results showed that the content of IAA may not be the major factor that changes the firmness of the two different watermelon lines. The ABA content of the two watermelon materials increased with ripening, but the ABA content of W1-1 was consistently higher than that of PI186490, with the most significant difference at 35 DAP.

The correlation analysis of watermelon flesh hardness and other indexes was conducted and is shown in Table 4: (1) watermelon flesh firmness had a significant correlation with chewiness and springiness, having stronger correlation with chewiness, while flesh hardness directly affected the taste of watermelon; (2) there was a strong correlation between flesh firmness and the cell wall components in watermelon, among which protopectin had the strongest correlation with a correlation coefficient of 0.787; and (3) watermelon flesh firmness was negatively correlated with IAA and ABA.

**TABLE 4:** Correlation analysis between flesh firmness and concentration of cell wall components, IAA, ABA in watermelon.

	**Hardness**	**Chewiness**	**Cohesiveness**	**Springiness**	**Resilience**	**Adhesiveness**	**Cellulose**	**Protopectin**	**Hemicellulose**	**IAA**	**ABA**
Hardness	1.000										
Chewiness	0.915**	1.000									
Cohesiveness	0.244	0.474**	1.000								
Springiness	0.811**	0.846**	0.431	1.000							
Resilience	0.366	0.571*	0.826**	0.695**	1.000						
Adhesiveness	0.155	0.087	–0.185	0.033	–0.265	1.000					
Cellulose	0.763**	0.661**	0.149	0.593**	0.257	–0.282	1.000				
Protopectin	0.787**	0.779**	–0.034	0.511*	0.119	0.119	0.559**	1.000			
Hemicellulose	0.598**	0.566*	0.502*	0.526*	0.450	–0.429	0.782**	0.318	1.000		
IAA	–0.444	−0.425*	0.179	–0.215	0.008	0.314	–0.399	−0.701**	–0.242	1.000	
ABA	–0.459	−0.478*	−0.519*	–0.351	–0.279	–0.230	–0.256	–0.236	−0.505*	–0.309	1.000

**Presents significant correlation at 0.01 level. *Presents significant correlation at 0.05 level.

### The Observation of Paraffin Sections of PI186490 and W1-1 Fruit During Ripening

**FIGURE 5:**
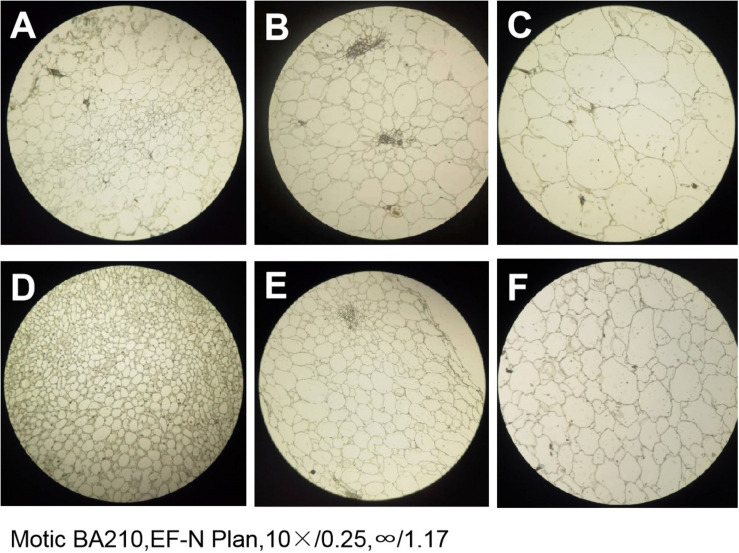
The paraffin section of W1-1 and PI186490 (10×). **(A)** W1-1 7DAP; **(B)** W1-1 21DAP; **(C)** W1-1 35DAP; **(D)** PI186490 7DAP; **(E)** PI186490 21DAP; **(F)** PI186490 35DAP. Three individual replicates were used.

The paraffin sections of the flesh of the two parents at different periods were compared and revealed that the size and arrangement of the flesh cells of the two parents were different. At any stage, the PI186490 cells in the same field of vision were more numerous, had a closer arrangement and a clearer cell wall edge than the W1-1 cells, and the cell wall of PI186490 was thicker than that of W1-1 visually (Figure 5). This may be one of the reasons for the difference in fruit firmness between the two watermelon varieties.

### Identification of DEGs

During the development and ripening of watermelon fruits, W1-1 and PI186490 showed significant differences in flesh firmness at the same developmental stage, and thus, it was very important to confirm their DEGs at the same developmental stage to study the differences in the mechanisms mediating watermelon flesh firmness. Before DEG analysis, the quality of RNA-Seq samples were evaluated by correlation analysis (Supplementary Figure S1).

**FIGURE 6:**
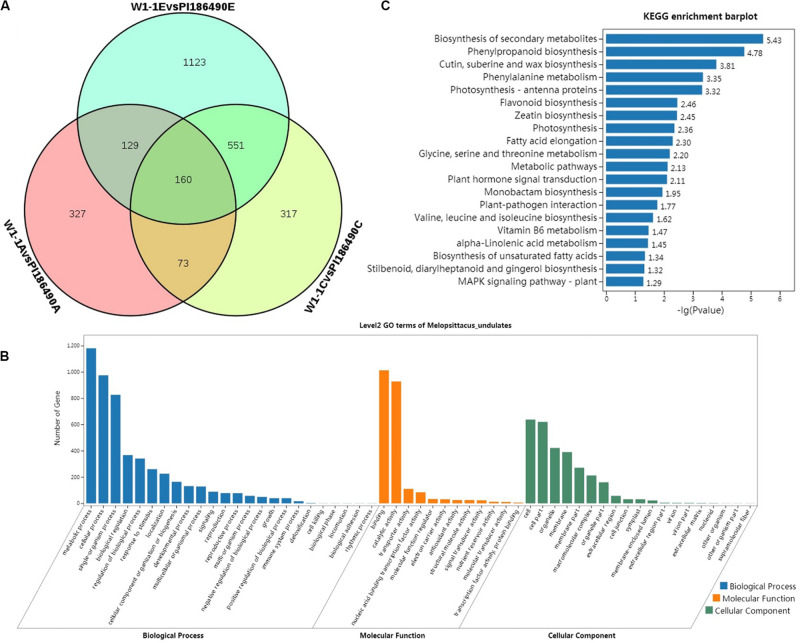
Differential analysis and enrichment analysis of transcriptome. Venn diagram of the DEGs of W1-1 and PI186490 fruits at three development stages (pollinated 7, 21, and 35 days) **(A)**, GO functional **(B),** and KEGG **(C)** enrichment analysis of the DEGs between W1-1 and PI186490 at three development stages (pollinated 7, 21, and 35 days).

As a result, a total of 11,405 DEGs were detected between W1-1 and PI186490, among which 3753 genes were annotated. Throughout the process of development and maturity of watermelon fruit, 160 annotated genes were significantly differentially expressed at three key developmental stages in the two experimental materials, and watermelon flesh hardness in these three stages also presented a significant difference, suggesting that these 160 DEGs may play a decisive role in the differences in watermelon flesh hardness (Figure 6A). Among them, there were eight genes related to plant growth hormone metabolism, namely, Cla016195 (ABA receptor), Cla005404 (9-cis-epoxycarotenoid dioxygenase), Cla015981 (1-aminocyclopropane-1-carboxylate oxidase), Cla023158(Ethylene-responsive transcription factor), Cla013991 (IAA-amido synthetase), Cla016785 (ethylene-responsive transcription factor), Cla022055 (Gibberellin receptor), and Cla015407 (gibberellin 3-beta-hydroxylase); five genes related to fruit maturation, Cla014096 (UDP-glycosyltransferase), Cla014189 (UDP-glycosyltransferase), Cla020325 (UDP-glycosyltransferase), Cla021220 (UDP-glycosyltransferase), and Cla002283 (endoglucanase); and five genes related to cell wall metabolism, Cla006266 (pectinesterase), Cla006576 (Hydroxycinnamoyl transferase), Cla007259 (Pectinesterase inhibitor), Cla007803 (Pectinesterase family protein), and Cla002573 (pectate lyase).

### GO and KEGG Functional Enrichment Analysis of DEGs

Gene Ontology function enrichment analysis was conducted on 3753 DEGs of W1-1 and PI186490 during the whole development and maturation process of watermelon fruit to understand the function distribution of these DEGs at the macro level. As shown in Figure 6B, in the GO biological process, the DEGs of W1-1 and PI186490 were divided into 24 GO terms, and there were many DEGs enriched in the terms metabolic process, cellular process and single-organism process, with 1180, 974, and 826 DEGs, respectively. The terms with the lowest number of DEGs were biological phase, cell killing, biological phase, locomotion, biological adhesion and rhythmic process, which all had only one DEG. In the GO molecular function category, the DEGs of W1-1 and PI186490 were divided into 12 GO terms, among which those containing a large number of DEGs included binding and catalytic activity, containing 1012 and 927 DEGs, respectively. In the GO cellular component category, the DEGs of W1-1 and PI186490 were divided into 19 GO terms, among which cell, cell part, organelle and membrane contained a larger number of DEGs.

To better understand the fruit hardness difference in the watermelon fruit varieties and the development of DEGs involved in the process of the main metabolic pathways, the DEGs of W1-1 and PI186490 enriched in KEGG pathways were evaluated to determine the metabolic pathways and signal transduction pathways in the KEGG database, and the first 20 pathways with the smallest P-value were selected for plotting (Figure 6C). The most significantly enriched pathway was biosynthesis of secondary metabolites (ko01110) with 144 DEGs, followed by phenylpropanoid biosynthesis (ko00940), cutin, suberin and wax biosynthesis (ko00073), and phenylalanine metabolism (ko00360) with 36, 10, and 15 DEGs, respectively. Lignin is an important product of phenylpropane metabolism in plants. It provides essential strength, hydrophobicity and resistance to the hostile environment to plant cell walls. The DEGs in these four metabolic pathways may play an important role in regulating the flesh firmness of watermelon.

The qRT-PCR results were showed in Supplementary Figure S2. Five genes were selected for qRT-PCR analysis of the samples of W1-1 and PI186490 fruits at three stages of development. It is found that the variation trend of gene relative expression measured by qRT-PCR is consistent with the variation trend of transcriptome sequencing data, indicating that transcriptome sequencing data and its subsequent analysis are reliable.

## Discussion

Different fruits have different flesh texture characteristics; it is difficult to quantify flesh texture artificially in sensory evaluations, but texture analyzers can be used to quantify texture parameters. The TPA results showed that there was a significant positive correlation between flesh hardness, chewiness, cohesiveness, springiness, and resilience of watermelon fruit, which was consistent with previous studies on pear (Meng et al., 2006), apple (Pan and Tu, 2005), and bayberry (Chen and Li, 2009) fruits. The TPA test is a simulation of the chewing process in the human mouth, and from the above results, flesh hardness, cohesiveness, resilience, springiness, and chewiness were closely related. Therefore, flesh hardness, cohesiveness, resilience, and springiness can comprehensively reflect the chewiness of flesh.

Cell wall metabolism is an important change related to flesh texture during fruit ripening (Sun et al., 2012a). The main components of the cell walls of fruit include pectin, cellulose and hemicellulose, which form an interwoven network structure with different kinds of modified cell wall proteins. The primary reason for fruit ripening and softening is the destruction of the internal structure of cell walls. The main manifestation is the separation of the flesh cells by the degradation of intercellular pectin substances (Goulao and Oliveira, 2008). Cellulose and hemicellulose play an important role in maintaining the stability of the cell wall structure. In this study, the pectin content of W1-1 and PI186490 increased first and then decreased with the development and ripening of watermelon fruit. The pectin content of PI186490 was always higher than that of W1-1, and the hardness of PI186490 was always higher than that of W1-1, which indicated that the change in the fruit flesh hardness was correlated with the pectin content. Many studies have shown that during the development and storage of fruits, the decrease in flesh firmness is often accompanied by an increase in the water-soluble pectin content (Fils-Lycaon and Buret, 1990; Duan et al., 2008; Wei et al., 2010; Gwanpua et al., 2014; Gao, 2018). However, in this experiment, only the total pectin content was determined, and the total pectin contains covalently bound pectin (CSP), water-soluble pectin (WSP) and ion-bound pectin (ISP); different forms of pectin have different effects on fruit quality, which may be the reason that the pectin content curve and hardness curve did not completely match in this test. In the study on the correlation between the pectin content and flesh hardness of Akiko pear (Huo et al., 2018), the total pectin content increased after fruit softening in three varieties and decreased after fruit softening in another three varieties, and thus, there were differences in the total pectin content after softening in different cultivars, which was consistent with the results of this experiment. Similar results were obtained for cellulose and hemicellulose, and the difference in cellulose and hemicellulose content is the reason for the difference in flesh hardness of different watermelon varieties, which was consistent with the research results of Liu et al. (2013) and Gao (2018).

Abscisic acid promotes the ripening of strawberry by upregulating the transcription levels of the PAL, DFR, PME, and PL genes, and IAA inhibits fruit ripening by downregulating the transcription levels of the PAL, DFR, PME, and PL genes (Lu, 2018). The study by Wang et al. (2017) showed that the ABA content in the fruits of 97103 (soft flesh) and PI179878 (hard flesh) increased during fruit development and ripening, and the ABA content of 97,103 was always higher than that of PI179878, similar to the results of our research. At the early stage of strawberry growth, auxin promoted fruit expansion; however, as fruit ripened, the auxin content decreased, and the ABA content increased (Ji et al., 2012).

Currently, there are few studies on the localization of watermelon flesh firmness. Juarez et al. (2013) used SNP markers to construct a genetic linkage map of an F_2_ isolated population, including 19 linkage groups, and located a major QTL related to watermelon flesh firmness on the 9th linkage group. Liu et al. (2014) located genes of edge flesh hardness and center flesh hardness on the 9th linkage group of watermelon in an F_2_ isolated population by resequencing two parents. Lu et al. (2016) located genes of edge flesh hardness on the 4th, 6th, and 8th linkage groups. Gao (2018) located the gene controlling watermelon flesh firmness in the 4.7 Mb physical interval of chromosome 6. In this study, the primary genes controlling watermelon flesh hardness were located in the physical interval of 1.53 Mb on chromosome 2 and 195 kb on chromosome 8, which was inconsistent with previous research results. It was speculated that because of the different watermelon materials, our results were also different.

Studies have found that MADS-box transcription factors regulate the biological processes of the transformation of plants from vegetative growth to reproductive growth, response to photoperiod, flowering time, formation of flower organs and pollen development (Huang et al., 2012). An increasing number of studies have shown that numerous MADS-box transcription factors are also involved in fruit development, maturation and cracking, regulation of photosynthesis and nutrient metabolism, and hormone signal transduction (Hu et al., 2005; Li et al., 2015; Liu et al., 2010). One MADS-box gene in tomato, *TOMATO AGAMOUS-LIKE 1 (TAGL1)*, is highly homologous to Arabidopsis *SHATTERPROOF (SHP)* and belongs to the AG subgroup, which is expressed in the ovule, carpel and developing pericarp (Busi et al., 2003; Hileman et al., 2006). Itkin et al. (2009) showed that the TAGL1 protein could bind to the promoter region of the *ACO1* and *ACS2* genes, providing a basis for the direct regulation of the MADS-box transcription factor in tomato fruits to ethylene biosynthesis genes. In addition, TAGL1 is involved in cell circulation regulation, flavonoid and lignin synthesis, and cuticle development (Garceau et al., 2017). The MADS-box is located in a vast network of fruit quality regulation, and the mechanism involved in fruit quality is relatively complex. The specific role of the MADS-box in the fruit maturity regulation network and its interaction with other factors have yet to be analyzed.

Xylan is the main hemicellulose in the secondary cell wall of plants. It accounts for approximately 30% of the dry weight of the cell walls of grasses and many woody plants (Gibeaut and Carpita, 1994). The reduction in xylan mutations in xylem secondary cell walls that lead to collapsed vessels suggests the importance of this polymer to cell wall strength (Brown et al., 2005; Zhong et al., 2005). GXMs (members of the DUF579 family) are glucuronoxylan methyltransferases catalyzing 4−*O*-methylation of glucuronic acid on xylan (Lee et al., 2012; Li et al., 2013; Urbanowicz et al., 2012). IRX15 and IRX15L, two DUF579 members, are involved in the biosynthesis and/or deposition of xylan in secondary cell walls (Brown et al., 2011; Jensen et al., 2011). In this study, the DUF579 family member Cla016033 was differentially expressed in the flesh of watermelon with different hardness, which may be related to xylan-related metabolism in the cell wall.

## Data Availability Statement

The datasets generated for this study can be found in the NCBI: PRJNA477364.

## Author Contributions

XW, LS, and FL conceived and designed the experiments. LS, LZ, TS, and YZ performed the field experiments. HC, CF, and CW performed the data analysis. LS wrote the manuscript. FL and XW reviewed drafts of the manuscript. All authors contributed to the manuscript and approved the submitted version.

## Conflict of Interest

The authors declare that the research was conducted in the absence of any commercial or financial relationships that could be construed as a potential conflict of interest.

## Footnotes

1 http://www.premierbiosoft.com/

2 http://www.cucurbitgenomics.org/
