# Supplementary material for: Linkage Mapping and Comparative Transcriptome Analysis of Firmness in Watermelon (Citrullus lanatus)
Source: Front Plant Sci. 2020 Jun 16;11:831. doi: 10.3389/fpls.2020.00831 (PMC7308538; doi:10.3389/fpls.2020.00831)
Supplement: Supplementary file 1 [file Presentation_1.pdf]

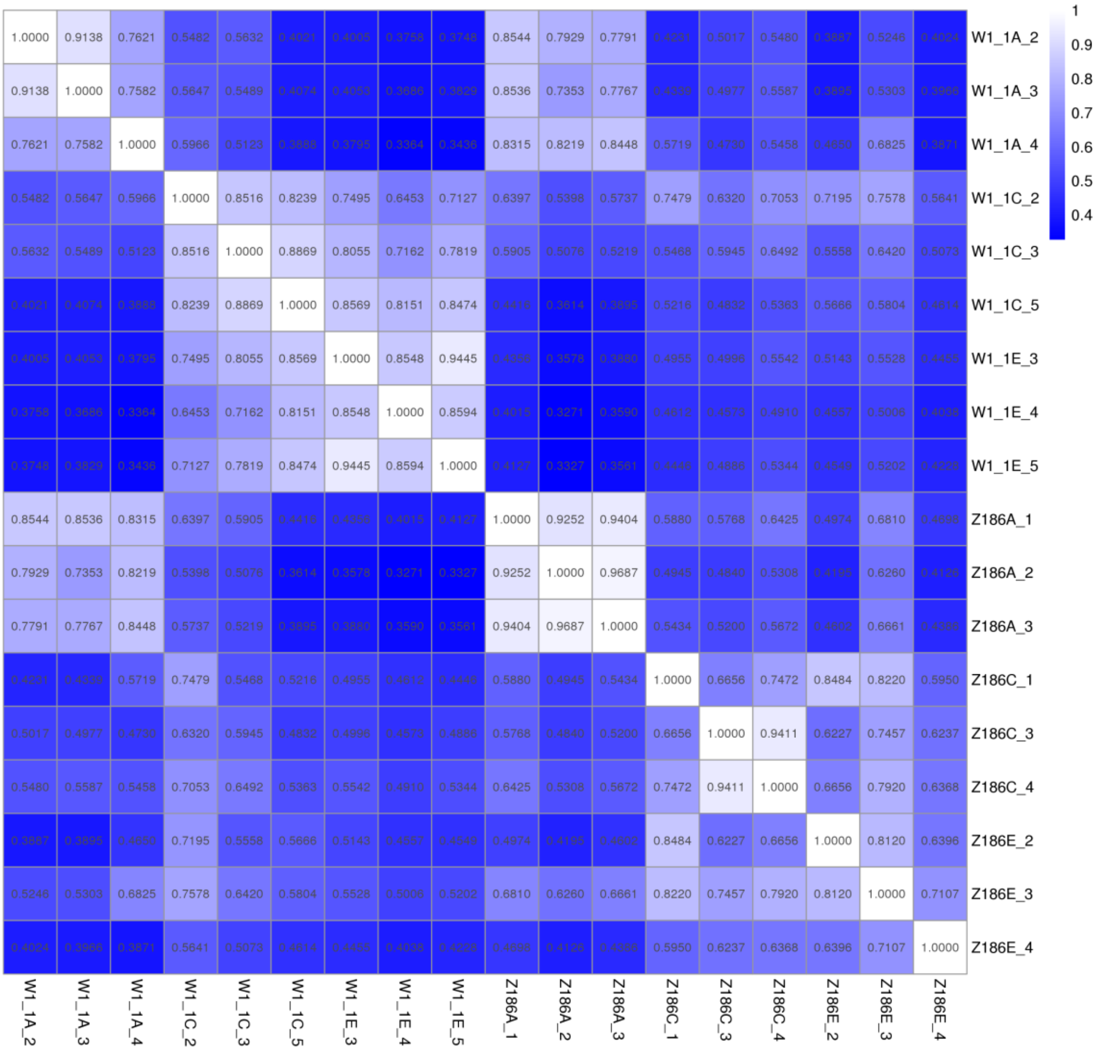

Fig.S1 Pearson correlation between samples

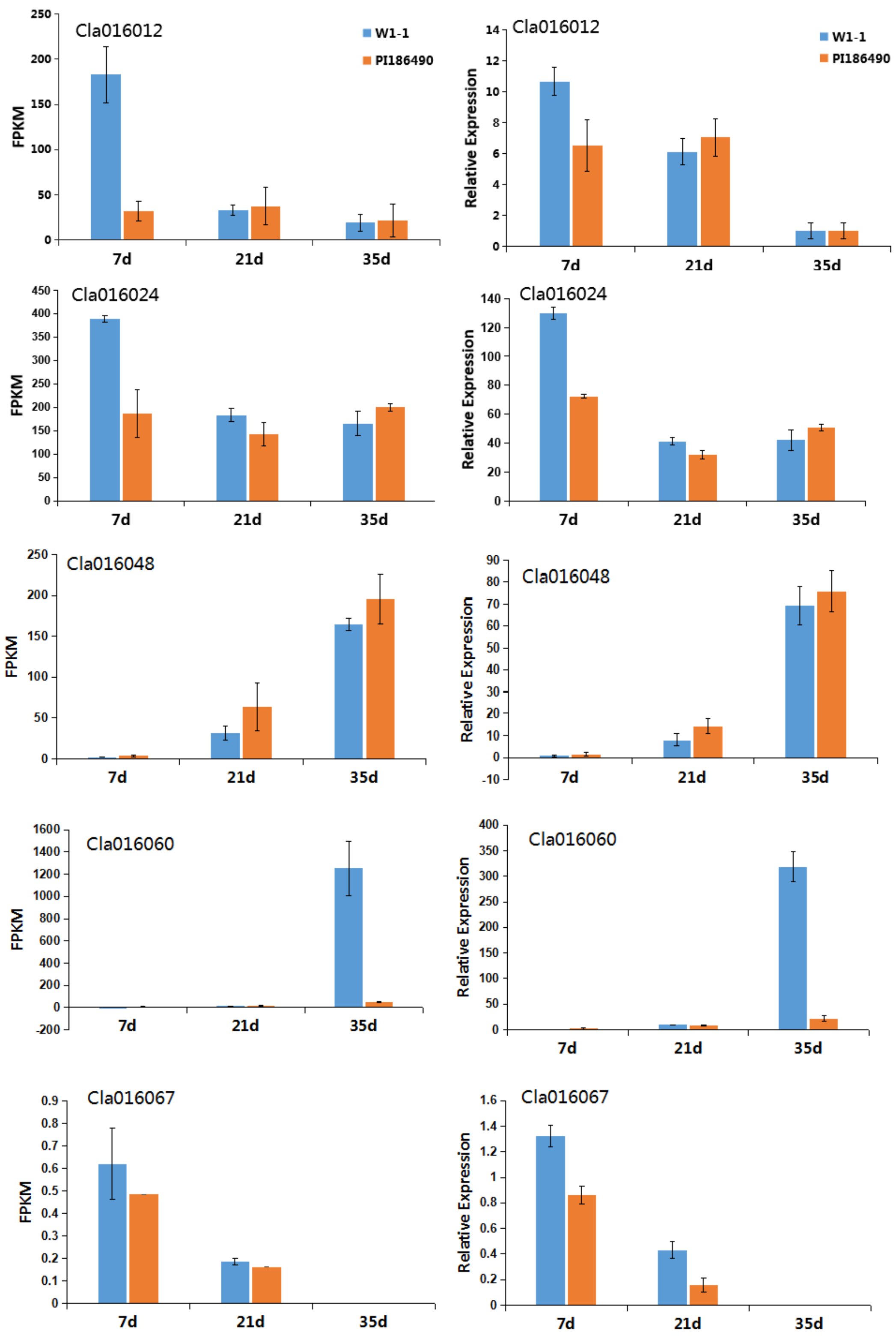

Fig.S2 Validation of genes by qRT-PCR analysis. The relative expression levels of five genes in three development stages of W1-1 and PI186490 by RNA-Seq using FPKM method and by qRT-PCR using the $2^{-\triangle\triangle CT}$ method. FPKM means Reads Per Kilobase of exon model per Million mapped reads. Bars represent mean ± SE (n=3). (Three biological replicates per sample).
